# Temporal trends in healthcare resource utilization and costs following acute myocardial infarction

**DOI:** 10.1186/s13584-020-0364-y

**Published:** 2020-02-12

**Authors:** Arthur Shiyovich, Harel Gilutz, Jonathan Eli Arbelle, Dan Greenberg, Ygal Plakht

**Affiliations:** 1 Department of Cardiology, Beilinson Hospital, Rabin Medical Center, Rabin Medical Center, 39 Jabotinski Street, 49100 Petah Tikva, Israel; 2 Sackler Faculty of Medicine, Tel Aviv University, Tel Aviv, Israel; 3 Faculty of Health Sciences, Ben-Gurion University of the Negev, Beer-Sheva, Israel; 4 Maccabi Health Services, Southern Region, Beer-Sheva, Israel; 5 Soroka University Medical Center, Beer-Sheva, Israel

**Keywords:** Acute myocardial infarction; healthcare resource utilization, Costs, Temporal trends

## Abstract

**Background:**

Acute myocardial infarction (AMI) is associated with greater utilization of healthcare resources and financial expenditure.

**Objectives:**

To evaluate temporal trends in healthcare resource utilization and costs following AMI throughout 2003–2015.

**Methods:**

AMI patients who survived the first year following hospitalization in a tertiary medical center (Soroka University Medical Center) throughout 2002–2012 were included and followed until 2015. Length of the in-hospital stay (LOS), emergency department (ED), primary care, outpatient consulting clinic visits and other ambulatory services, and their costs, were evaluated and compared annually over time.

**Results:**

Overall 8047 patients qualified for the current study; mean age 65.0 (SD = 13.6) years, 30.3% women. During follow-up, LOS and the number of primary care visits has decreased significantly. However, ED and consultant visits as well as ambulatory-services utilization has increased. Total costs have decreased throughout this period. Multivariate analysis, adjusted for potential confounders, showed as significant trend of decrease in LOS and ambulatory-services utilization, yet an increase in ED visits with no change in total costs.

**Conclusions:**

Despite a decline in utilization of most healthcare services throughout the investigated decade, healthcare expenditure has not changed. Further evaluation of the cost-effectiveness of long-term resource allocation following AMI is warranted. Nevertheless, we believe more intense ambulatory follow-up focusing on secondary prevention and early detection, as well as high-quality outpatient chest pain unit are warranted.

## Introduction

Survivors of acute myocardial infarction (AMI) continue to be at greater risk and utilize more healthcare resources, resulting in increased economic burden [1–3]. Moreover, expenditure throughout the first year following an AMI seems to be exceptionally high, estimated between $22,000–$87,000 (European countries and the United States) [3–6]. Most aspects of AMI management have experienced dramatic changes throughout recent years, including patient characteristics, presentation, invasive procedures, medical therapy, and outcomes which overall have improved significantly [1, 7]. However, data dealing with healthcare resource utilization and expenditure are sparse and inconsistent [8, 9]. The aim of the current study was to evaluate the temporal trends in healthcare resource utilization and costs following AMI.

## Methods

### Study population

The current study included patients who survived the first year following hospitalization with an AMI (index admission) throughout 1.1.2002–31.12.2012 in Soroka University Medical Center and who were members of one of the two largest insurers in Israel (Health Maintenance Organizations – HMOs): Maccabi Healthcare Services or (Maccabi) or Clalit Health Services (Clalit). For patients who suffered more than one AMI throughout the investigated period, the first event was considered as the index event.

Soroka University Medical Center is a tertiary referral center (~ 1200 beds), serving the metropolitan area of Beer-Sheva, Negev, southern Israel. This region comprises approximately 60% of the area of Israel and is inhabited by more than 500,000 residents, Jews over 60%. Thus, this hospital and its cardiology department face unique challenges: for example management of acute myocardial infarction transferred after thrombolytic therapy in Eilat (the most southern city in Israel), while all other cardiology departments in Israel can provide primary PCI services for STEMI.

The local ethics committees of the participating organizations approved the study, which was performed consistently with the Helsinki declaration.

### Individual follow-up and study outcomes

The personal follow-up of the study participants began 1 year after the index admission (i.e. 1 year survivors) and lasted up to10 years (or until the participant’s death). The follow-up period of the study ended in 31.12.2015. Evaluation of healthcare services utilization comprised the following: the length of in-hospital stay, all causes, number of emergency department (ED) visits not resulting in hospital admissions, number of primary care and outpatient clinic visits and other ambulatory services (e.g. various ambulatory diagnostic or therapeutic procedures). In addition, the per-patient total cost was estimated by summing the costs of all the utilized services.

### Data sources and classifications

Data were retrieved from the participating organizations’ computerized medical records. The baseline data obtained from the hospitals’ database included patient characteristics clinical evaluation and management as previously reported for the Soroka Acute Myocardial Infarction (SAMI-II) project [10]. Most variables were obtained using The International Classification of Diseases, Ninth Revision, Clinical Modification (ICD-9-CM) discharge codes (see Appendix for examples). Furthermore, the diagnosis of anemia was grouped together with low hematocrit and low hemoglobin blood levels at discharge as following: for men—hemoglobin < 13 g/dL and hematocrit < 39%; for women—hemoglobin < 12 g/dL and hematocrit < 36%. Similarly, the diagnosis of renal diseases included high creatinine blood levels (at discharge) ≥1.2 mg/dL. The diagnosis of diabetes mellitus comprised high levels of Hemoglobin A1C (greater than 6.5%). Diagnoses of diabetes mellitus (DM) with complications were classified in accordance with the target of complications. In this way, the diagnoses of DM with renal manifestations were defined as renal diseases, the diagnoses of DM with peripheral circulation manifestations were grouped with peripheral vascular diseases. The remaining diagnoses of DM were assigned to DM with no recorded complications.

Adherence with medical therapy for the following guideline-recommended medications: Aspirin, statins, beta blockers and angiotensin-converting-enzyme inhibitors (ACEi) or Angiotensin II receptor blockers (ARBs). The adherence rate was calculated based on the issued prescriptions during the first year following hospital discharge and calculated consistently with the well described proportion of days covered (PDC) parameter [11]. Adherence (PDC) of 80% or more for all the evaluated medication groups was considered as adherent [12–15].

Utilization of the various healthcare services were obtained from the records of the insurers. Per-patient costs were obtained for the year before the index admission and for each year throughout the follow-up period thereafter. Costs were based on the rates set by the Israeli Ministry of Health. Costs were obtained in local currency (Israeli Shekel – [ILS]) and converted to United States Dollar (USD) based on currency exchange rates (1 ILS = 0.29 USD, 04.01.2020). In this context it should be mentioned, that primary clinic visits mainly refers to a physician-patient encounter while ambulatory visit includes various ambulatory procedures (analyses or treatments) which are charged differently by the HMOs.

Mortality data were obtained from the Ministry of the Interior Population Registry.

Patient-level data from the records of the above-mentioned authorities were linked through the individual personal identification number.

### Statistical analysis

Statistical analyses were performed with IBM SPSS Statistics 25 software. Baseline characteristics of the study cohort were presented as mean and standard deviation (SD) for continuous variables and n/percent for categorical data. Healthcare service utilization and their costs were calculated for every year throughout 2003–2015 and are presented as mean and SD. Comparisons of the investigated outcomes between the years throughout the study period were performed using Analysis of variance (ANOVA) test for linear trend in the univariate level. In addition, generalized estimating equations (GEE) were built separately for each investigated outcome, for the purpose of controlling for repeated measures for each patient. Three level models for each outcome were built. The first level model (“unadjusted”) included the variable of the study year only. The second level model included the variable of the study year and the personal time following the AMI (follow-up period). The third level model included all the above variables in addition to the patients’ baseline characteristics, healthcare service utilization 1 year before AMI and adherence with medical therapy during the first year after AMI. The results of the models are presented as the coefficients (B) with Standard errors (SE) and 95% confidence intervals (CI). For each test, two-sided *p* < 0.05 was considered significant.

## Results

### Baseline characteristics

**Fig. 1:**
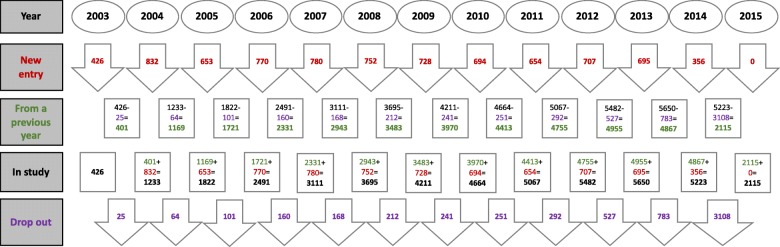
The study flowchart: number of patients by the study year

Throughout 2002–2012 a total of 12,535 patients were admitted with an AMI and screened for the current study. Overall 8047 patients qualified for the study (mean age 64.96 ± 13.58), as the rest were excluded for the following reasons: 2293 (18.3%) were not insured by the HMOs participating in the study, 930 (8.9%) died during the index admission, amongst the survivors of the index AMI an additional 1265 (13.2%) died during the first follow-up year and thus were excluded from the study. A flowchart displaying the number of patients as well as inclusion and exclusion for each year is presented in Fig. 1. Baseline characteristics of the patients by study year are presented in Table 1.

**Table 1:** The baseline characteristics of the study population by study year

Year	2003	2004	2005	2006	2007	2008	2009	2010	2011	2012	2013	2014	2015	p for trend
n	426	1233	1822	2491	3111	3695	4211	4664	5067	5482	5650	5223	2115	
*Demographics*														
Age at AMI, years; Mean (SD)	64.20 (12.97)	64.09 (12.96)	63.76 (12.88)	63.40 (12.84)	63.06 (12.72)	63.02 (12.88)	62.68 (12.94)	62.44 (12.93)	62.23 (12.95)	62.09 (13.00)	61.99 (13.01)	61.78 (12.97)	60.79 (12.74)	< 0.001
Sex, Male	293 (68.8)	850 (68.9)	1278 (70.1)	1761 (70.7)	2235 (71.8)	2662 (72.0)	3068 (72.9)	3404 (73.0)	3717 (73.4)	4013 (73.2)	4165 (73.7)	3886 (74.4)	1579 (74.7)	< 0.001
Muslims	85 (20.0)	246 (20.0)	371 (20.4)	508 (20.4)	642 (20.6)	774 (20.9)	910 (21.6)	1056 (22.6)	1180 (23.3)	1306 (23.8)	1367 (24.2)	1310 (25.1)	515 (24.3)	< 0.001
*Cardiac diseases*														
Supraventricular arrhythmias	46 (10.8)	159 (12.9)	221 (12.1)	306 (12.3)	373 (12.0)	456 (12.3)	497 (11.8)	540 (11.6)	570 (11.2)	616 (11.2)	645 (11.4)	577 (11.0)	223 (10.5)	0.003
CHF	80 (18.8)	186 (15.1)	261 (14.3)	341 (13.7)	390 (12.5)	450 (12.2)	499 (11.8)	525 (11.3)	533 (10.5)	554 (10.1)	555 (9.8)	486 (9.3)	172 (8.1)	< 0.001
Pulmonary heart disease	13 (3.1)	56 (4.5)	88 (4.8)	140 (5.6)	162 (5.2)	214 (5.8)	241 (5.7)	282 (6.0)	307 (6.1)	314 (5.7)	349 (6.2)	308 (5.9)	109 (5.2)	0.012
CIHD	305 (71.6)	932 (75.6)	1407 (77.2)	1928 (77.4)	2458 (79.0)	2951 (79.9)	3426 (81.4)	3860 (82.8)	4205 (83.0)	4565 (83.3)	4761 (84.3)	4447 (85.1)	1820 (86.1)	< 0.001
AV block	14 (3.3)	42 (3.4)	68 (3.7)	92 (3.7)	111 (3.6)	118 (3.2)	129 (3.1)	148 (3.2)	159 (3.1)	177 (3.2)	183 (3.2)	161 (3.1)	63 (3.0)	0.092
*Cardiovascular risk factors*														
Renal diseases	206 (48.4)	479 (38.8)	640 (35.1)	844 (33.9)	1001 (32.2)	1125 (30.4)	1244 (29.5)	1335 (28.6)	1416 (27.9)	1499 (27.3)	1447 (25.6)	1296 (24.8)	504 (23.8)	< 0.001
Diabetes Mellitus	157 (36.9)	409 (33.2)	598 (32.8)	801 (32.2)	1028 (33.0)	1250 (33.8)	1416 (33.6)	1536 (32.9)	1678 (33.1)	1802 (32.9)	1908 (33.8)	1770 (33.9)	681 (32.2)	0.825
Dyslipidemia	291 (68.3)	815 (66.1)	1236 (67.8)	1671 (67.1)	2140 (68.8)	2555 (69.1)	2969 (70.5)	3331 (71.4)	3680 (72.6)	4006 (73.1)	4170 (73.8)	3871 (74.1)	1563 (73.9)	< 0.001
Hypertension	200 (46.9)	648 (52.6)	929 (51.0)	1252 (50.3)	1556 (50.0)	1872 (50.7)	2114 (50.2)	2368 (50.8)	2575 (50.8)	2796 (51.0)	2921 (51.7)	2670 (51.1)	1074 (50.8)	0.253
Obesity	87 (20.4)	255 (20.7)	369 (20.3)	535 (21.5)	683 (22.0)	849 (23.0)	1001 (23.8)	1145 (24.5)	1254 (24.7)	1368 (25.0)	1414 (25.0)	1302 (24.9)	553 (26.1)	< 0.001
Smoking	168 (39.4)	487 (39.5)	770 (42.3)	1064 (42.7)	1359 (43.7)	1617 (43.8)	1896 (45.0)	2110 (45.2)	2313 (45.6)	2524 (46.0)	2616 (46.3)	2451 (46.9)	1030 (48.7)	< 0.001
PVD	58 (13.6)	165 (13.4)	245 (13.4)	305 (12.2)	365 (11.7)	411 (11.1)	444 (10.5)	471 (10.1)	492 (9.7)	501 (9.1)	472 (8.4)	416 (8.0)	164 (7.8)	< 0.001
Family history of IHD	18 (4.2)	60 (4.9)	109 (6.0)	169 (6.8)	225 (7.2)	292 (7.9)	351 (8.3)	419 (9.0)	469 (9.3)	512 (9.3)	543 (9.6)	531 (10.2)	237 (11.2)	< 0.001
*Other disorders*														
COPD	20 (4.7)	79 (6.4)	121 (6.6)	155 (6.2)	182 (5.9)	206 (5.6)	220 (5.2)	239 (5.1)	247 (4.9)	261 (4.8)	273 (4.8)	235 (4.5)	87 (4.1)	< 0.001
Neurological disorders	47 (11.0)	139 (11.3)	207 (11.4)	277 (11.1)	321 (10.3)	399 (10.8)	465 (11.0)	508 (10.9)	570 (11.2)	623 (11.4)	640 (11.3)	563 (10.8)	210 (9.9)	0.841
Anemia	199 (46.7)	576 (46.7)	836 (45.9)	1140 (45.8)	1414 (45.5)	1737 (47.0)	1975 (46.9)	2215 (47.5)	2409 (47.5)	2578 (47.0)	2670 (47.3)	2440 (46.7)	944 (44.6)	0.530
GI bleeding	6 (1.4)	20 (1.6)	35 (1.9)	48 (1.9)	60 (1.9)	61 (1.7)	73 (1.7)	78 (1.7)	84 (1.7)	94 (1.7)	97 (1.7)	84 (1.6)	35 (1.7)	0.391
Schizophrenia/Psychosis	5 (1.2)	18 (1.5)	22 (1.2)	40 (1.6)	50 (1.6)	59 (1.6)	59 (1.4)	62 (1.3)	63 (1.2)	62 (1.1)	65 (1.2)	54 (1.0)	17 (0.8)	0.001
Alcohol/drug addiction	9 (2.1)	20 (1.6)	30 (1.6)	48 (1.9)	67 (2.2)	76 (2.1)	79 (1.9)	90 (1.9)	96 (1.9)	106 (1.9)	105 (1.9)	97 (1.9)	37 (1.7)	0.743
Malignancy	8 (1.9)	34 (2.8)	45 (2.5)	56 (2.2)	69 (2.2)	87 (2.4)	97 (2.3)	98 (2.1)	106 (2.1)	119 (2.2)	128 (2.3)	121 (2.3)	43 (2.0)	0.430
*Administrative characteristics of the hospitalization*														
Admitted/transposed to ICCU	288 (67.6)	869 (70.5)	1339 (73.5)	1848 (74.2)	2352 (75.6)	2797 (75.7)	3233 (76.8)	3635 (77.9)	3963 (78.2)	4264 (77.8)	4401 (77.9)	4101 (78.5)	1707 (80.7)	< 0.001
Length of hospital stay (days), > 7	232 (54.5)	666 (54.0)	986 (54.1)	1375 (55.2)	1668 (53.6)	1924 (52.1)	2127 (50.5)	2308 (49.5)	2439 (48.1)	2594 (47.3)	2599 (46.0)	2339 (44.8)	909 (43.0)	< 0.001
Type of AMI, STEMI	323 (75.8)	816 (66.2)	1188 (65.2)	1531 (61.5)	1826 (58.7)	2087 (56.5)	2331 (55.4)	2545 (54.6)	2750 (54.3)	2961 (54.0)	2966 (52.5)	2722 (52.1)	1130 (53.4)	< 0.001
*Results of echocardiography*														
*Echocardiography performance*	328 (77.0)	974 (79.0)	1482 (81.3)	2054 (82.5)	2620 (84.2)	3117 (84.4)	3606 (85.6)	4043 (86.7)	4421 (87.3)	4784 (87.3)	4945 (87.5)	4624 (88.5)	1904 (90.0)	< 0.001
Severe LV dysfunction	31 (9.5)	91 (9.3)	135 (9.1)	176 (8.6)	210 (8.0)	234 (7.5)	244 (6.8)	264 (6.5)	291 (6.6)	332 (6.9)	358 (7.2)	346 (7.5)	117 (6.1)	< 0.001
LV hypertrophy	24 (7.3)	52 (5.3)	67 (4.5)	84 (4.1)	102 (3.9)	125 (4.0)	143 (4.0)	150 (3.7)	143 (3.2)	163 (3.4)	162 (3.3)	146 (3.2)	65 (3.4)	< 0.001
Mitral regurgitation	13 (4.0)	37 (3.8)	61 (4.1)	82 (4.0)	100 (3.8)	113 (3.6)	144 (4.0)	169 (4.2)	200 (4.5)	222 (4.6)	211 (4.3)	176 (3.8)	61 (3.2)	0.717
Tricuspidal regurgitation	8 (2.4)	19 (2.0)	31 (2.1)	44 (2.1)	53 (2.0)	73 (2.3)	80 (2.2)	90 (2.2)	100 (2.3)	115 (2.4)	118 (2.4)	100 (2.2)	39 (2.0)	0.571
Pulmonary hypertension	13 (4.0)	37 (3.8)	59 (4.0)	91 (4.4)	119 (4.5)	159 (5.1)	182 (5.0)	211 (5.2)	225 (5.1)	234 (4.9)	247 (5.0)	211 (4.6)	78 (4.1)	0.418
*Results of angiography*														
*Angiography performance*	238 (55.9)	795 (64.5)	1260 (69.2)	1731 (69.5)	2250 (72.3)	2749 (74.4)	3225 (76.6)	3666 (78.6)	4039 (79.7)	4418 (80.6)	4646 (82.2)	4362 (83.5)	1774 (83.9)	< 0.001
Measure of CAD, No or non-significant	7 (2.9)	42 (5.3)	62 (4.9)	85 (4.9)	105 (4.7)	125 (4.5)	150 (4.7)	173 (4.7)	196 (4.9)	215 (4.9)	240 (5.2)	216 (5.0)	75 (4.2)	< 0.001
One vessel	50 (21.0)	177 (22.3)	279 (22.1)	412 (23.8)	554 (24.6)	705 (25.6)	832 (25.8)	958 (26.1)	1083 (26.8)	1205 (27.3)	1277 (27.5)	1236 (28.3)	534 (30.1)	< 0.001 < 0.001
Two vessels	71 (29.8)	221 (27.8)	358 (28.4)	496 (28.7)	653 (29.0)	788 (28.7)	902 (28.0)	1041 (28.4)	1143 (28.3)	1255 (28.4)	1325 (28.5)	1262 (28.9)	506 (28.5)	
Three vessels/ LM	110 (46.2)	355 (44.7)	561 (44.5)	738 (42.6)	938 (41.7)	1130 (41.1)	1340 (41.6)	1494 (40.8)	1617 (40.0)	1743 (39.5)	1804 (38.8)	1648 (37.8)	659 (37.1)	
Type of treatment, Noninvasive	179 (42.0)	418 (33.9)	533 (29.3)	687 (27.6)	770 (24.8)	841 (22.8)	868 (20.6)	862 (18.5)	879 (17.3)	899 (16.4)	833 (14.7)	691 (13.2)	258 (12.2)	
PCI	203 (47.7)	673 (54.6)	1070 (58.7)	1506 (60.5)	1960 (63.0)	2360 (63.9)	2725 (64.7)	3079 (66.0)	3391 (66.9)	3707 (67.6)	3908 (69.2)	3669 (70.2)	1512 (71.5)	< 0.001
CABG	44 (10.3)	142 (11.5)	219 (12.0)	298 (12.0)	381 (12.2)	494 (13.4)	618 (14.7)	723 (15.5)	797 (15.7)	875 (16.0)	908 (16.1)	862 (16.5)	345 (16.3)	
*Healthcare utilization an year before AMI; Mean (SD)*														
Hospitalizations LOS, days	4.28 (7.74)	4.61 (9.79)	4.47 (10.45)	4.38 (9.81)	4.1 (8.49)	4.04 (8.47)	3.73 (7.79)	3.59 (7.49)	3.49 (8.34)	3.27 (8.02)	3.03 (7.90)	2.8 (7.76)	2.82 (8.27)	< 0.001
Number of ED visits	0.02 (0.17)	0.03 (0.20)	0.03 (0.20)	0.03 (0.19)	0.02 (0.19)	0.02 (0.17)	0.02 (0.17)	0.03 (0.20)	0.06 (0.32)	0.11 (0.52)	0.13 (0.49)	0.16 (0.55)	0.12 (0.47)	< 0.001
Number of primary clinic visits	3.01 (5.50)	6.97 (8.45)	8.20 (9.04)	9.11 (9.45)	9.65 (9.85)	10.03 (10.06)	10.23 (10.12)	10.37 (10.14)	10.48 (10.2)	10.50 (10.16)	10.72 (9.97)	10.76 (9.91)	10.41 (9.90)	< 0.001
Number of ambulatory clinic visits	5.18 (8.52)	5.15 (10.64)	3.83 (8.60)	4.79 (10.86)	5.39 (12.00)	5.92 (12.39)	6.12 (12.00)	6.59 (14.07)	6.86 (14.96)	6.83 (14.51)	6.64 (13.27)	6.70 (13.08)	6.61 (12.45)	< 0.001
Number of consulting clinic visits	0.53 (1.25)	0.78 (1.68)	0.82 (1.70)	0.93 (1.85)	0.94 (1.83)	0.97 (1.81)	0.98 (1.83)	1.02 (1.92)	1.03 (1.92)	1.04 (1.95)	1.06 (1.96)	1.06 (1.93)	1.05 (2.02)	< 0.001
Total cost, USD	2391 (4029)	2962 (5066)	2909 (4878)	3055 (5516)	3090 (5457)	3136 (5458)	3043 (4819)	3109 (5231)	3208 (6453)	3124 (6182)	3006 (5675)	2922 (5585)	2966 (6618)	0.996
Follow-up														
Full adherence to medical treatment during the 1st year	64 (15.0)	218 (17.7)	362 (19.9)	553 (22.2)	724 (23.3)	888 (24.0)	1065 (25.3)	1204 (25.8)	1293 (25.5)	1354 (24.7)	1378 (24.4)	1275 (24.4)	529 (25.0)	< 0.001
Age in the present year, years; Mean (SD)	64.20 (12.97)	64.74 (12.95)	65.00 (12.88)	65.11 (12.84)	65.23 (12.71)	65.64 (12.82)	65.77 (12.88)	66.00 (12.85)	66.27 (12.84)	66.55 (12.83)	66.65 (12.83)	66.69 (12.86)	67.40 (12.64)	< 0.001
Period since AMI, years; Mean (SD)	1.00 (0)	1.33 (0.47)	1.85 (0.74)	2.28 (1.05)	2.71 (1.34)	3.16 (1.62)	3.61 (1.90)	4.08 (2.17)	4.56 (2.44)	4.97 (2.74)	5.14 (2.87)	5.37 (2.75)	6.68 (2.53)	< 0.001

The data are presented as n (%) unless otherwise stated. Dyslipidemia is defined as recorded diagnosis of dyslipidemia and/or average LDL level > 130 mg/dL during the hospitalization. Anemia is defined as recorded diagnosis of anemia and/or hemoglobin level < 14 g/dL or hematocrit level < 42% for males and hemoglobin level < 12 g/dL or hematocrit level < 37% for females during a hospitalization

Abbreviations: *AMI* Acute myocardial infarction; *AV* Atrioventricular; *CABG* Coronary artery bypass surgery; *CAD* Coronary artery disease; *CHF* Congestive heart failure; *CIHD* Chronic ischemic heart disease; *COPD* Chronic obstructive pulmonary disease; *ED* emergency department; *GI* Gastro-intestinal; *ICCU* Intensive Cardiac Care Unit; *IHD* Ischemic heart disease; *LOS* length of (hospital) stay; *LM* Left main (artery); *LV* Left ventricular; *PCI* Percutaneous coronary intervention; *PVD* Peripheral vascular disease; *SD* standard deviation; *STEMI* ST segment elevation myocardial infarction; *USD* United States Dollars

Throughout the later study years, patients’ age was lower and the rate of male patients was greater compared with earlier years. The prevalence of most traditional cardiovascular risk factors increased (except diabetes mellitus) while the prevalence of most other investigated comorbidities decreased over time. Throughout the later period the rates of STEMI - ST segment elevation myocardial infarction (STEMI) and of 3-vessel or left main coronary artery disease decreased as well. However, the rate of invasive treatments (Percutaneous coronary intervention or/and Coronary artery bypass surgery [PCI/CABG]) has increased. The rate of adherence with medical therapy during the first year after AMI has improved significantly over time.

### Trend of healthcare resources utilization and costs after AMI

**Fig. 2:**
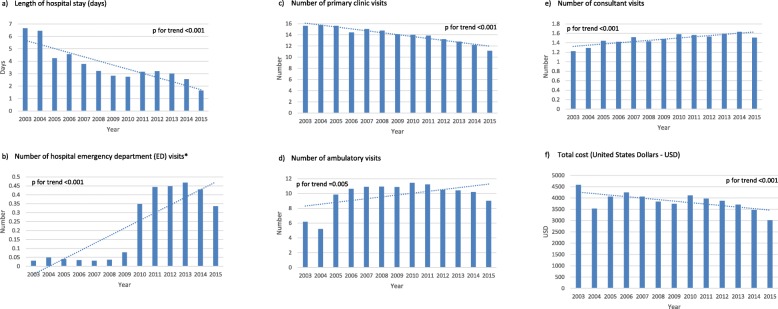
Trends of healthcare resources utilization and costs during the study period, by study year. **a** Length of hospital stay (days). **b** Number of hospital emergency department (ED) visits (with no hospital admission). **c** Number of primary clinic visits. **d** Number of ambulatory visits. **e** Number of consultant visits. **f** Total cost (United States Dollars - USD)

Healthcare resource utilization and costs by study year are displayed in Fig. 2. Throughout the investigated decade the mean length of in-hospital stay and the number of primary care visits decreased significantly (6.65 days 2003 vs. 1.63 days 2015 and 15.6 vs. 11.1 respectively; p for trend < 0.001 for each). During the same period, the mean number of ED visits, consultant visits and ambulatory visits increased significantly (0.03 vs. 0.34, p for trend < 0.001; 1.2 vs. 1.5, p for trend < 0.001; 6.2 vs. 9.0, p for trend = 0.005 respectively). Mean total costs decreased throughout the investigated period (4579 vs. 3018 USD, p for trend < 0.001).

### Multivariable analysis

The results of the multivariable models for each investigated outcome are presented in Table 2. The first level models (“unadjusted”) show an increased number of ED, ambulatory and consulting clinic visits, while the yearly length of hospital stay, number of primary care visits and the annual total cost decreased during the study period. The second level model, adjusting for time following AMI, shows an increase in the number of ED visits and a decrease in all other parameters. Finally, the third level model which included all variables in addition to patients’ baseline characteristics, healthcare service utilization 1 year before AMI and adherence with medical therapy during the first year after AMI, shows a significant decrease in the length of hospital stay, primary care visits and consultant visits, while the yearly number of ED visits has increased and no significant change was observed in the ambulatory visits. No statistically significant difference was found in the adjusted total costs in the third level model.

**Table 2:** Trends of healthcare resources utilization and costs – multivariate analysis

Dependent variable	Model Level^a^	B (SE)^c^	95% CI (B)	p
Length of hospital stay (days per patient-year)	1	− 0.075 (0.008)	(−0.091; − 0.060)	< 0.001
	2	− 0.155 (0.009)	(− 0.172; − 0.137)	< 0.001
	3	−0.142 (0.030)	(− 0.201; − 0.083)	< 0.001
ED visits (number of visits per patient-year)^b^	1	0.276 (0.009)	(0.259; 0.293)	< 0.001
	2	0.277 (0.021)	(0.236; 0.318)	< 0.001
	3	0.289 (0.014)	(0.262; 0.317)	< 0.001
Primary clinic visits (number of visits per patient-year)	1	−0.358 (0.026)	(−0.409; − 0.307)	< 0.001
	2	− 0.696 (0.026)	(− 0.746; − 0.645)	< 0.001
	3	−0.612 (0.025)	(− 0.661; − 0.563)	< 0.001
Ambulatory visits (number of visits per patient-year)	1	0.009 (0.004)	(0.001; 0.018)	0.033
	2	−0.012 (0.005)	(−0.021; − 0.003)	0.008
	3	0.008 (0.005)	(−0.001; 0.016)	0.091
Consultant visits (number of visits per patient-year)	1	0.015 (0.003)	(0.008; 0.021)	< 0.001
	2	−0.026 (0.004)	(−0.034; − 0.019)	< 0.001
	3	− 0.025 (0.004)	(− 0.033; − 0.017)	< 0.001
Total cost (USD per patient-year)	1	−0.015 (0.005)	(− 0.025; − 0.006)	0.001
	2	− 0.050 (0.006)	(− 0.061; − 0.040)	< 0.001
	3	−0.005 (0.011)	(− 0.027; 0.016)	0.627

^a^ The first level models – unadjusted. The second level models - adjusted for the time following the Acute myocardial infarction (AMI). The third level models - adjusted for the time following the AMI, age (at the evaluated year), sex, nationality, utilization / cost during an year before AMI, adherence to medical treatment during the 1st year, type of AMI, type of treatment and comorbidities (renal diseases, diabetes mellitus, neurological disorders, anemia, supraventricular arrhythmias, Congestive heart failure, Chronic obstructive pulmonary disease and Alcohol/drug addiction

^b^ Emergency department (ED) visits with no hospital admission

^c^ Beta coefficient represents the degree of change in the outcome variable for every 1-unit of change in the predictor variable i.e. for every increase of 1 year throughout the follow-up period (e.g. 2003–2004 or 2014–2015) the LOS / cost differed by B units (day / USD), on average

Abbreviations: *B* regression coefficient; *CI* confidence interval; *SE* standard error; *USD* United States Dollars

## Discussion

The current study investigated temporal trends in healthcare resource utilization throughout more than a decade (2003–2015) among post-AMI patients. Main findings include: throughout the investigated period utilization of most health-care resources, particularly the length of hospital stay and primary clinic visits have decreased. However, a major increase in ED visits was observed. The unadjusted healthcare expenditure has decreased throughout the study period. When this analysis is adjusted for potential confounders no statistically significant change in total cost was observed.

It has been widely described that AMI survivors are prone to increased morbidity (compared to matched general population), healthcare services utilization and economic burden which seem to be exceptionally high throughout the first year after the AMI followed by some subsequent decline [2–5, 16]. We found a significant attenuation in the length of hospitalizations and ambulatory services utilization throughout the last decade yet an increase in the rate of ED visits and no change in total cost. This finding is consistent with the findings of Chen et al. [17] that showed a decrease in hospitalization of AMI patients (for heart failure) in Medicare beneficiaries 1998–2010 and with those of Chaudhry et al. [18] who reported a decline in the rate of recurrent AMI hospitalization between 1999 and 2000. Likosky et al. [9],who investigated readmission rate within the first year following AMI, found no difference throughout 1998–2008. Importantly, our analysis includes all hospitalizations rather than those for a particular cause, while previous reports showed that about 30% of readmissions are for reasons unrelated to the original condition [19]. In partial consistency with our findings, a recent report from the US found significant differences across the country and between different services, in ambulatory and outpatient resource utilization and expenditure following hospital discharge with an AMI (31–180 days) between 1999 and 2014 [20].

The current study was not designed to determine the causes for the observed time dependent changes. However, several reasons can be suggested to explain the decline in hospitalizations and primary medicine services utilization. First and foremost, introduction of evidence based treatments in the acute phase management incorporated into AMI guidelines, improvements in the timeliness, and in particularly the use of invasive revascularization treatments as also observed in the current study [21, 22]. Furthermore, significant improvements have been reported in secondary prevention treatments and post discharge management including control of risk factors, the use of guideline recommended medications and rehabilitation [18, 23]. Improved post-discharge adherence with guideline recommended therapies, as observed in the current study, is also an important factor which has previously been reported to be associated with reduced healthcare resources utilization [14]. It should be mentioned that individual long-term healthcare resource utilization tends to decrease after the first year post AMI [16]. However, this individual decline does not seem to be a prominent explanation for the observed trend in the current study, since an adjustment for the time from the index AMI (the second level models) was performed.

A trend of increase in ED visits was observed throughout the years, consistent with previous reports [20]. This disparity in the trends of ED utilization and the other resources could possibly be attributed to increased awareness and caution by the AMI survivors and their healthcare providers to complaints that could be related to an acute cardiac condition. Alternatively insufficient availability of out-of-hospital services could all also cause increased frequency of ED visits [24]. Furthermore, the exceptionally greater increase in the ED visits overtime should be a focus of further investigation and intervention; for example, dedicated high quality out of hospital services with utilization of high sensitivity troponin point-of-care essays or the establishment of chest pain unit for such patient. In addition, a trend of increased age, increased prevalence of cardiovascular risk factors and other comorbidity were reported among AMI patients. The latter trends could counter-balance a trend of reduction and support a trend of increase in healthcare resources (e.g. ED visits) utilization [25, 26].

Total annual healthcare expenditure found in this study is lower than that reported in other studies [9–12]. This disagreement is probably due to differences in healthcare and insurance systems and the methodology for estimating healthcare expenditure. The unadjusted (the first level model) and the adjusted to time from AMI (the second level model), total cost has shown a significant trend of decrease throughout the evaluated decade. However, following adjustment to multiple potential confounders in a multivariate model (the third level model) we did not find statistically significant differences in overall healthcare expenditure throughout the investigated time period. The latter findings probably mean that the unadjusted decrease in costs is explained by the changes in patient characteristics, administered treatments (e.g. increase in interventional therapy) and improvement in adherence with medical therapy. A previous report, [9] found an increase of 16.5% in the healthcare expenditure per AMI patient between the years 1998–2008 in the US. Interestingly, the authors found that expenditures for skilled nursing facilities, hospice, home health agency, durable medical equipment, and outpatient care nearly doubled during the period of 31–365 days after admission. The differences can stem from different study periods, adjustment to confounders as well as differences in healthcare systems. A recent report that evaluated expenditure on Medicare beneficiaries 180 days following AMI in four time periods 1999–2000, 2004, 2008 and 2013–2014) found that expenditure increased 13.9% from 1999 to 2000, and 2013,-2014, but declined 0.5% between 2008 and 2013–2014 [20]. It should however be mentioned that excluding patients that died throughout the first year might have resulted in underestimation of costs since treating these patients is usually associated with increased expenditure [16]. This possibly had an impact over the temporal trend of costs, especially when considering the trend of improvement in short term survival observed during the study period [7].

Our findings should be discussed considering several important characteristics of the Israeli health care system. Israel has a national health insurance financed predominantly via progressive taxation and includes four competing, non-profit health plans (HPs) which provide a broad package of services determined by the government [27, 28]. The HPs provide care in the community and purchase hospital services for their members. Nevertheless, approximately 40% of health care expenditure is financed privately. Access to primary care physicians and community-based specialists is good overall (travel and waiting times), although in some areas and populations (e.g. Israeli Arabs) it could be insufficient resulting in excessive use of hospital services. The occupancy rates of Israeli hospitals is one of the highest among the Organization for Economic Co-operation and Development (OECD) countries (greater than 90%) while the length of stay is one of the shortest (around 4 days) [27, 28]. Furthermore, hospital pricing system is considered outdated and limits their reimbursement while pricing of novel procedures and technologies and rising.

## Limitations

The current study has several limitations. First, the study is retrospective and based mostly on administrative data (e.g. ICD-9-CM codes) hence prone for bias resulting from such a design such as recording bias. Furthermore, such a design also does not enable identification of the causes for the observed trends. Second, patients from only one hospital with unique characteristics, population mix and from a unique region in Israel were evaluated hence generalizability is limited. Third, differentiation of cardiovascular from non-cardiovascular related healthcare services utilization was not performed. Fourth, out of pocket expenses were not evaluated thus true costs are probably higher. Fifth, it is possible that unaccounted changes, not directly related to the AMI, in patients’ health throughout the individual follow-up might have led to changes in health care utilization.

## Conclusions and implications

The current study investigated temporal trends in healthcare resource utilization and costs in post-AMI patients throughout 2003–2015. The study shows a significant decrease in the length of the in-hospital stay and ambulatory services utilization with an increase in ED visits. Total costs (unadjusted or adjusted for the time following AMI), have declined. However, following adjustment for a variety of potential confounders no significant trend was found. Further and more comprehensive studies evaluating such trends, mechanisms and causes behind the observed findings, as well as the association between hospital services utilization and ambulatory utilization of healthcare services and costs in general and in subgroups of interest, are warranted, in order to improve resource allocation, patient outcomes and maximize cost-effectiveness in the long-term management of AMI patients. Nevertheless, even with current findings we believe that more resources and attention should be shifted towards ambulatory follow-up focusing on secondary prevention measures (i.e. optimal control of risk factors and prudent, cost-effective, early detection of subsequent and recurrent manifestations of atherosclerosis), particularly in high risk patients. Furthermore, we recommend reinforcing out of hospital measures for high quality chest pain evaluation such as a dedicated service, for chest pain evaluation (e.g. outpatient chest pain units) with high sensitivity troponin point-of-care essays and non-invasive coronary evaluation tests that would reduce ED visits and in-hospital evaluations.

## Data Availability

Please contact author for data requests.

## Appendix

### Appendix 1. Diagnoses and interventions according to ICD-9-CM codes

Coronary artery bypass graft (CABG) Z361–Z362;

Thrombolytic therapy and/or Percutaneous coronary intervention (PCI): PCI Z3601–Z3607;

Renal diseases 403*, 580–591*, 7910, V420, 2504*;

Obesity 278, 2780*;

Gastro-intestinal hemorrhage 53,021, 5307, 53,082, 5310*, 5314*, 5316*, 5322*, 5324*,5326*, 5330*, 5322*, 5334*, 5336*, 5340*, 5342*, 5344*,5346*, 53,501, 53,511, 53,521, 53,531, 53,541, 53,551,53,561, 53,783, 53,784, 56,202, 56,203, 56,212, 56,213,56,985, 578*;

Anemia 280–285*;

Pulmonary heart disease 416*-417*;

Chronic obstructive pulmonary disease 491*, 492*, 494*, 496*;

Malignant neoplasm 148–208*, 230–233*;

Alcohol or drug addiction 303–3050*, 3052–3059*, V113;

Neurological disorders 294*, 310*, 320–349*, 430–438*, Z0131*;

Schizophrenia and/or psychosis 295*, 298*, V110.
